# Comparison of volume and frequency advancement feeding protocols in very low birth weight neonates

**DOI:** 10.12669/pjms.341.14092

**Published:** 2018

**Authors:** Afaq Hussain, Abdur Rehman, Nazia Fatima

**Affiliations:** 1 Dr. Afaq Hussain, FCPS (Paeds Med), Trainee, Children Hospital Complex and the Institute of Child Health, Multan, Pakistan; 2 Dr. Abdur Rehman, FCPS (Paeds Med) FCPS (Neonatal Paeds Med), Assistant Professor and Head of Neonatal ICU, Children Hospital Complex and the Institute of Child Health, Multan, Pakistan; 3 Dr. Nazia Fatima, FCPS (Paeds Med), Trainee, Children Hospital Complex and the Institute of Child Health, Multan, Pakistan

**Keywords:** Very low birth weight neonates, Feeding methods

## Abstract

**Objective:**

To determine the outcomes in very low birth weight (VLBW) neonates receiving volume advancement versus frequency advancement feeding protocols.

**Methods:**

This controlled clinical trial was conducted in Children Hospital Multan within duration of 6 months from February 2017 to August 2017. VLBW neonates having weight < 1500 g at the time of birth were included. The protocol for frequency advancement (FA) group was to give 1 ml/kg human or pre-formula milk after every 8 hours and in volume advancement (VA) group after every 3 hours initially. After three days, in FA group duration of feeds was decreased gradually from 8 to 2 hours and feed volume of 10 ml.kg^-1^.day^-1^ until full-recommended dose of feeding i.e. 150 ml.kg^-1^.day^-1^ reached. While in VA group, volume of 20 ml.kg^-1^.day^-1^ was given until full-recommended dose of feeding reached. Days to achieve full feed, weight gain, and length of hospital stay were primary study outcomes.

**Results:**

Baseline weight of neonates was 1148 (111) grams in VA 1179 (106) grams in FA groups (p-value 0.18). In VA group, full feed was achieved in 11.04 (2.38) days versus 15.76 (2.48) days in FA group (P-value <0.001). Duration of IV fluid therapy were 13.5 (8.4) days in FA group versus 9.4 (7.6) in VA group (p-value <0.001). Moreover weight gain at the end of feeding protocol was significantly higher in VA group 1440 (78) grams versus 1284 (99) grams in FA group (P-value <0.001). Necrotizing entero-colitis occurred in only one neonate that was belonging to volume advancement group.

**Conclusion:**

Volume advancement (VA) feeding is better as compared to frequency advancement (FA) feeding in very low birth weight neonates.

## INTRODUCTION

Advancements in practice and experience of neonatologists have greatly reduced the death rate of premature neonates born with very low birth weight (VLBW). In these VLBW neonates, the optimal growth rate is still unknown.^1^ According to European society guidelines, the growth rate of these neonates should be equivalent to intra-uterine fetal growth rate.^2^ Failure to achieve this optimal growth rate results in growth retardation at the time of discharge and even this growth retardation can persist during childhood and adult life.^3^-^5^ Slow growth of these VLBW infants results in impaired neuro-motor and cognitive outcomes.^6^,^7^ It also have adverse effects on metabolic and cardio-vascular function.^8^,^9^ Therefore, the only way to prevent these problems is optimal nutritional supply to these VLBW infants so that growth rate equal to intra-uterine fetal growth can be achieved.

Human breast milk is the highly recommended nutritional product in infants for at least six months of age.^10^ However, researches have concluded that standard doses of breast milk given to pre-term or VLBW neonates did not fulfill their metabolic demands and may result in growth retardation.^11^ In developed countries, human milk is fortified with extra nutrition supplements usually extracted from cow's milk.^12^ A systematic review has concluded that fortified breast milk results in higher neonatal growth as compared to breast milk alone in early-hospitalized period.^13^ On the other hand, in developing countries, these nutrient-enriched formulations are not readily available due to their high cost. So mother's breast milk and pre-term formula milk are routinely used to fulfill high nutritional requirements of VLBW neonates. However some studies have suggested that a higher amount of breast milk (> 200 ml/kg/day) as compared to the normal dose (150 ml/kg/day) of breast milk is needed in these infants but others say that there is no need for extra dose.^14^,^15^ There are two recommended methods of starting the feeding in these infants, one method is that to start feeding earlier and increase the feeding volumes rapidly to achieve recommended daily dose and the other is increasing the frequency of feeds rather than increasing the volume of feeds. Both of these protocols have some advantages and disadvantages. In this study, we compared the volume versus frequency bases protocols of feeding in VLBW neonates in a tertiary care neonatal care facility.

## METHODS

This controlled clinical trial was conducted in Children Hospital Multan within duration of 6 months from February 2017 to August 2017. VLBW neonates having weight < 1500 g at the time of birth were included. We included 90 neonates in this study initially, 3 patients lost the follow up so final analysis included 87 neonates. However, neonates having congenital anomalies, chromosomal abnormalities, gastro-intestinal malformations, necrotizing entero-colitis and sepsis were excluded from analysis. Informed consent from parents of newborns and ethical approval from hospital ethical committee was taken.

VLBW neonates were divided using random number tables into volume advancement and frequency advancement groups. In all neonates, minimal enteral feeding (to prime GIT) was started after twenty four (24) hours of birth and continued for three days either using breast milk or pre-term formula milk. The protocol for frequency advancement (FA) group was to give 1 ml/kg human or pre-formula milk after every 8 hours and in volume advancement (VA) group after every 3 hours.^16^ After three days, in FA groups the frequency of feeds was decreased gradually from 8 hours to 2 hours with a minimum increase in feed volume of only 10 ml.kg^-1^.day^-1^ until full-recommended dose of feeding i.e. 150 ml.kg^-1^.day^-1^reached. While in VA group, there was an increase in volume of 20 ml.kg^-1^.day^-1^ until full-recommended dose of feeding i.e. 150 ml.kg^-1^.day^-1^ reached. In all neonates, total parenteral nutrition was started within 24 hours after birth to provide additional nutrients to meet the metabolic requirements. Protein intake was started at 2.0 g.kg^-1^.day^-1^ to 3 g.kg^-1^.day^-1^ and then 4 g.kg^-1^.day^-1^ if tolerated by the neonate. Intra-lipids were also given to these neonates to fulfill daily caloric intake. Intra-lipids were stopped after achieving 15% of total neonatal feed in both groups. While protein intake was stopped after achieving 50% of total neonatal feed. Feeding was given through naso-gastric (NG) tube initially until the babies corrected gestational age crossed 34 weeks or when the baby weight was > 1700 grams, then oral feeding was started.

Days to achieve full feed, weight gain, and length of hospital stay were primary study outcomes. All neonates were kept in the hospital until their weight reaches 2000 grams and they were medically fit to be discharged from the hospital. We kept these neonates in the hospital for a longer period even after achieving the full feed to monitor the outcomes of study. Because most of the families were belonging from far area and it was very difficult to monitor baby weight after discharge.

For data analysis, we used SPSS v23. Total weight gain, days to reach full feed and hospital stay between the groups was compared using independent sample t-test.

## RESULTS

Mean gestational age of neonates was almost same in volume advancement (VA) and frequency advancement (FA) groups. Baseline weight of neonates was 1148 (111) grams in VA 1179 (106) grams in FA groups (p-value 0.18). There were 24 (54.5%) male neonates in VA group and 23 (53.5%) male neonates in FA group (p-value 0.92). APGAR score after 5 minutes of birth was 8.31 (0.98) in VA group and 8.32 (0.96) in FA group (p-value 0.96). Table-I.

**Table-I:** Baseline characteristics of neonates.

Variable	VA Group (N=44)	FA Group (N=43)	P-value
Gestational Age (weeks)	29.72 (1.37)	29.76 (1.71)	0.90
Baseline Weight (Grams)	1148 (111)	1179 (106)	0.18
Male Gender (%)	24 (54.5%)	23 (53.5%)	0.92
Female Gender (%)	20 (45.5%)	20 (46.5%)	
APGAR score after 5 mins of Birth	8.31 (0.98)	8.32 (0.96)	0.97

There were 17 (38.6%) neonates in VA group in whom mother's breast milk was given and 18 (41.9%) in FA group in whom mother's breast milk was given for needing purpose while remaining neonates received pre-term formula milk. Time to reach full feed was significantly shorter in VA group as compared to FA group. In VA group, full feed was achieved in 11.04 (2.38) days while in FA group full feeding was achieved in 15.76 (2.48) days (P-value <0.001). Duration of IV fluid therapy were also prolonged in FA group 13.5 (8.4) days in versus 9.4 (7.6) in VA group (p-value <0.001). Moreover weight gain at the end of feeding protocol was significantly higher in VA group 1440 (78) grams versus 1284 (99) grams in FA group (P-value <0.001). Necrotizing entero-colitis occurred in only one neonate that was belonging to volume advancement group. That neonate was kept NPO for seven days after developing NEC and feeding was established again and the baby started tolerating the feed. Duration of hospital stay was similar between the groups (Table-II).

**Table-II:** Study outcome variables.

Variable	VA Group (N=44)	FA Group (N=43)	P-value
Mother's Breast Milk (%)	17 (38.6%)	18 (41.9%)	0.75
Formula Milk (%)	27 (61.4%)	25 (58.1%)	
Days to Reach Full Feed	11.04 (2.38)	15.76 (2.48)	<0.001
Days of IV fluids	9.4 (7.6)	13.5 (8.4)	<0.001
Weight at the end of feeding protocol (Grams)	1440 (78)	1284 (99)	<0.001
Weight at the time of Discharge (Grams)	2085 (63)	2072 (56)	0.29
Hospital Stay (Days)	41.20 (7.93)	42.62 (5.94)	0.34

## DISCUSSION

In our study, we found no significant adverse effects of volume advancement in VLBW neonates. There was less time taken to reach full feeds in VA group and less duration of IV fluids requirements in VA group. Weight gain at the end of feeding protocol was also significantly less in FA group 1284 (99) versus 1440 (78) grams in VA group. However, total weight gain at the time of discharge was same between the groups; 2072±56 grams in VA group and 2085±63 grams in FA group. Total duration of hospital stay was also similar between the groups. We did not found any significant adverse effects in volume advancement group. There was only neonate in the whole study in whom NEC occurred that neonate was in VA group, but this single incidence has no significant importance. Recent systematic reviews have also concluded that VA did not increases the risk of NEC in neonates and is a safe option.^17^,^18^

In our study, we started enteral feeding after 24 hours of birth. Studies have concluded that increasing the duration of parenteral feeding and delaying the enteral feeding is associated with prolonged hospital stay and have no effects on NEC prevention.^19^ Furthermore, parenteral nutrition also increase the risk of catheter related blood stream infections.^20^ Bombell et al.^21^ concluded that early enteral feeding increases the GIT motility, prevent normal flora and reduces the risk of infections. In our study, the total duration of IV fluids requirements was 9.4 (7.6) days in VA group and 13.5 (8.4) in FA group i.e. less in VA group. Therefore, volume advancement also have favorable outcomes for preventing infections. However, we did not found any incidence of infections in our study neonates.

Karagol et al.^22^ also found less time to reach full feed and shorter duration of IV fluid requirements in VA group similar to our study. Moreover, in their study days to weight gain was also rapid in VA group. Krishnamurthy et al. also found similar results regarding weight gain in VA group neonates.^23^

Like our study, Zubani et al.^16^ also did not found any significant difference regarding hospital stay and total weight at the time of discharge between the groups. Caple et al.^24^ have also found similar results. Recent Cochrane database review have concluded that VA feeding is better than FA feeding because it takes less time to reach full feed, shorter duration of parenteral feeds and rapid weight gain. In addition, it has no adverse effects on NEC.^25^ Furthermore, some researchers have found that VA feeding also has beneficial effects on the development of neural outcomes.^26^,^27^

## CONCLUSION

Volume advancement (VA) feeding is better as compared to frequency advancement (FA) feeding in very low birth weight neonates.

### Authors' Contribution

**AH:** Conceived, wrote the manuscript, takes the responsibility and is accountable for all aspects of the work in ensuring that questions related to the accuracy or integrity of any part of the work are appropriately investigated and resolved.

**AR:** Did data Analysis, helped in writing the manuscript, and did review.

**NF:** Did data collection and review.
